# HIV/AIDS Drugs for Sub-Saharan Africa: How Do Brand and Generic Supply Compare?

**DOI:** 10.1371/journal.pone.0000278

**Published:** 2007-03-14

**Authors:** Colleen V. Chien

**Affiliations:** Fenwick & West LLP, Oakland, California, United States of America; University of Sydney, Australia

## Abstract

**Background:**

Significant quantities of antiretroviral drugs (ARVs) to treat HIV/AIDS have been procured for Sub-Saharan Africa for the first time in their 20-year history. This presents a novel opportunity to empirically study the roles of brand and generic suppliers in providing access to ARVs.

**Methodology/Principal Findings:**

An observational study of brand and generic supply based on a dataset of 2,162 orders of AIDS drugs for Sub-Saharan Africa reported to the Global Price Reporting Mechanism at the World Health Organization from January 2004-March 2006 was performed. Generic companies supplied 63% of the drugs studied, at prices that were on average about a third of the prices charged by brand companies. 96% of the procurement was of first line drugs, which were provided mostly by generic firms, while the remaining 4%, of second line drugs, was sourced primarily from brand companies. 85% of the generic drugs in the sample were manufactured in India, where the majority of the drugs procured were ineligible for patent protection. The remaining 15% was manufactured in South Africa, mostly under voluntary licenses provided by brand companies to a single generic company. In Sub-Saharan African countries, four first line drugs in the dataset were widely patented, however no general deterrent to generic purchasing based on a patent was detected.

**Conclusions/Significance:**

Generic and brand companies have played distinct roles in increasing the availability of ARVs in Sub-Saharan Africa. Generic companies provided most of the drugs studied, at prices below those charged by brand companies, and until now, almost exclusively supplied several fixed-dose combination drugs. Brand companies have supplied almost all second line drugs, signed voluntary licenses with generic companies, and are not strictly enforcing patents in certain countries. Further investigation into how price reductions in second line drugs can be achieved and the cheapest drugs can actually be procured is warranted.

## Introduction

An aggressive drive to increase access to antiretroviral drugs (ARVs) by HIV-infected patients in developing countries is underway. An unprecedented level of resolve and funding, channeled through the Global Fund to treat AIDS, Malaria, and Tuberculosis (the Global Fund), and the U.S. President's Emergency Plan for AIDS Relief (PEPFAR), has been directed at various treatment targets, the most ambitious of which is the G8 goal of universal access by 2010 [1].

Although more than drugs will be needed to reach these goals, scaling up treatment will require a reliable and affordable supply of ARVs. However, to date, comprehensive empirical data on the characteristics and determinants of this supply are scarce. While a number of reports have focused on price [2]–[3], an understanding of the role of brand and generic suppliers is currently lacking. This understanding could be used to inform strategies to increase the availability of affordable and appropriate ARVs. Such strategies are critical to bolstering the short-term credibility of scale up efforts and long-term sustainability of treatment as drugs must be taken for the lifetime of a patient, and donor funds such as the Global Fund have already faced shortfalls [4].

Comparisons between brand and generic procurement of ARVs also have relevance to questions about how access to patented medicines by patients in developing countries can be achieved. To encourage generic production of patented medicines, the rights of countries to practice patented inventions without patentholder permission–through a practice called “compulsory licensing”–have been affirmed and expanded through the Doha Declaration and the permanent amendment of a core agreement of the World Trade Organization [5]. Yet there has been little empirical analysis of the extent to which these and other mechanisms have actually encouraged generic supply.

Since December 2003, the World Health Organization (WHO) has collected transaction data about purchases of ARVs for developing countries through the Global Price Reporting Mechanism (GPRM) [6]. This dataset presents the opportunity to do an observational study of the procurement of brand and generic HIV/AIDS drugs and consider its implications for scaling up.

## Methods

Data on 2,162 orders of ARVs in oral solid (adult) formulation for Sub-Saharan Africa from January 2004 to March 2006 were obtained from the GPRM. The GPRM tracks ARV procurement of UN organizations, the Global Fund, and government and NGO purchasers [7]. WHO estimates that GPRM orders represented 50% of the total procurement of ARVs for Sub-Saharan Africa in 2005 (written communication with WHO). The GPRM reports the total number of units transacted and the quantity of compound per unit. Quantities of compound were converted into patient-year equivalents using WHO dosing guidelines [8]. Prices were reported exclusive of taxes, tariffs, and transportation costs, and drug donations were excluded from the analysis. Orders were coded as “generic” or “brand” based on the manufacturer listed on each order. Additional information on the regulatory, patent, voluntary license, and price discount status of various compounds was obtained from published accounts. Information on oral and powder (child) formulations was not included in the analysis due to the difficulty of calculating patient-year equivalent quantities associated with variations in pediatric weight.

## Results

### Supplier and Price Patterns

Sixty-three percent of the volume of the ARVs in the dataset was ordered from generic companies, and 37% from brand companies (Table 1). Brand prices were on average three times more expensive than generic prices (Table 1), however supply patterns for individual drugs and drug segments varied widely (Table S1). In addition, price variation across countries and orders was observed.

**Table 1:** First and Second Line Antiretroviral Drugs Procured for Sub-Saharan Africa

Drug Name	Volume (patient year equivalents)	% of Total Volume	Percentage Brand	Percentage Generic	Avg. Brand Price*	Avg. Generic Price*
First Line ARVs	522,517	96%	35%	65%	277	114
Second Line ARVs	18,984	4%	93%	7%	591	601
Total	541,501	100%	37%	63%	304	116

N = 2,162 orders

Volumes calculated on the basis of WHO daily dosing guidelines to generate patient year equivalents

* Average prices in $/patient yr and calculated on the basis of total $s paid for drugs/total drugs in category.

### First v. second line drugs

In single and combination form, five drugs dominated the procurement studied, representing 96% of the ARVs ordered (Table 1). These five drugs–stavudine (d4T), zidovudine (AZT), lamivudine (3TC), nevirapine (NVP) and efavirenz (EFV)–formed the first line regimen recommended by the WHO during the time of the dataset [8]–[9]. For the purposes of this analysis, they are referred to as “first line” while others are referred to as “second line.” Second line compounds, used in the event of treatment failure, comprised the remaining 4% of drugs in the dataset (Table 1).

While generic companies provided more first line drugs than did brand companies, 93% of second line drugs was provided by brand companies (Table 1). In addition, first and second line drugs had significantly different price patterns. First line brand drugs were consistently more costly than first line generic drugs (Table S1), on average two to three times more expensive ($277/patient yr brand price vs. $114/ patient yr generic price, Table 1) In contrast, average brand and generic prices for second line drugs were roughly equal, with generic prices actually slightly higher than brand prices on average (Table 1).

### Fixed Dose Combinations

Differences were also seen in generic and brand supply patterns of multiple compound or “fixed dose” combination drugs. Favored for their simpler compliance and supply requirements, fixed dose formulations comprised a third of the procurement studied, the single combination of stavudine, lamivudine, and nevirapine accounting for 20% of the total (Table 2). Three of the top four fixed dose combinations were supplied entirely by generic companies, and combined molecules owned by different brand companies (Table 2). In contrast, the fourth fixed dose combination (AZT+3TC), supplied by both generic and brand companies, combined molecules of a single brand company, GlaxoSmithKline (Table 2).

**Table 2:** First Line Fixed Dose Combination Antiretroviral Drugs

Fixed Dose Combinations	Volume (patient year equivalents)	% of Total Volume	Percentage Brand	Percentage Generic	Brand Maker of Individual Drugs in Combination*
Stavudine (d4T)+Lamivudine (3TC)+Nevirapine (NVP)	109,971	20%	0%	100%	BMS+GSK+BI
Zidovudine (AZT)+Lamivudine (3TC)	61,847	11%	50%	50%	GSK
Zidovudine (AZT)+Lamivudine (3TC)+Nevirapine (NVP)	8,006	1%	0%	100%	GSK+BMS
Stavudine (d4T)+Lamivudine (3TC)	7,537	1%	0%	100%	GSK+BMS
Total	187,361	34%	17%	83%	-

N = 501 Orders

* BMS = Bristol Myers Squibb, GSK = GlaxoSmithKline, BI = Boehringer Ingelheim

### Patents

Patents confer the right to exclude others from making, using, selling, offering to sell, or importing an invention and are domestic in nature. An analysis of drug patent status in countries where drugs were made, as well as used, is therefore warranted. 85% of the generic drugs in the dataset were made in India for export and distribution in Sub-Saharan African countries, with the remainder manufactured in South Africa (Table 3). Except for drugs made and consumed in South Africa, the generic drugs in the dataset were imported for subsequent distribution into Sub-Saharan African countries. A brand company holding a patent in either a supplier or consumer jurisdiction could potentially block the generic production, import, sale, offer to sale, or use of a patented drug.

**Table 3:** Sources of Generic Antiretroviral Drugs

Country of Manufacture	India	South Africa
Generic Volume (patient years)	288,439	51,947
% of Total Generics Volume	85%	15%
% of Total Volume	53%	10%

### Supplier Country Patent Situation

Under Indian patent law, only drug compounds whose earliest date of patent application or “priority” date falls after January 1, 1995 are eligible for patent product protection [10]. The priority date status of first line drugs in the dataset is reported in Table 4. The priority dates associated with all individual first line as well as the majority of second line drugs in the dataset precede the critical date. (Second line drugs that have patents with a pre-January 1, 1995 priority date include abacavir, didanosine, ritonavir, saquinavir, nelfinavir, indinavir; the second line drugs that have patents with post-January 1, 1995 priority date are tenofovir, tenofovir/emtricitabine, lopinavir/ritonavir, and zidovudine/lamuviduine/abacavir [11]–[12].) Thus, basic versions of the overwhelming majority of drugs in the dataset were not eligible for brand company patent product protection in India. However, India's revised patent law offers protection to later-developed formulations, drug combinations, and drug compounds with priority dates after January 1, 1995 [10].

**Table 4:** Patent and Access Characteristics of First Line Antiretroviral Drugs in Sub-Saharan African Countries

Drug Name	Post-Jan 1, 1995 Priority Date?^a^	Number of Sub-Saharan African Countries in which drug patented b	Percentage of Total Countries^c^	Percentage of Order Volume from Generic Source	
				Countries in which drug patented^d^	Countries in which drug not patented^d^
Zidovudine (AZT)+Lamivudine (3TC)	Yes	32	84%	54%	46%
Lamivudine (3TC)	No	28	74%	62%	68%
Nevirapine (NVP)	No	25	66%	48%	59%
Zidovudine (AZT)	No	16^e^	42%	79%	74%
Stavudine (d4T)	No	1	3%	0%	58%
Efavirenz (EFV)	No	1	3%	0%	16%

a Source of data: [11], [12]

b Source of data: [14] The status of certain patents may have changed since publication, due to the failure to pay renewal fees, for instance. This would lend further support to the apparent brand company shift away from enforcement of patents in Sub-Saharan Africa.

c Total Countries = 38 countries in which transactions reported

d Calculation performed on the basis of countries that had transactions in that drug category (n = 24-31 countries). Number of countries in which drug patented with 0% generic purchases = 5 (AZT+3TC), 2 (3TC), 2 (NVP), 2 (AZT), 1 (d4T), 1 (EFV).

e The expiry date of the US patent on AZT was September 2005 [11].

The generic drugs from South Africa in the dataset were primarily sourced from a single firm, Aspen Pharmacare Ltd., which obtained voluntary licenses from a number of brand companies to make drugs generically. These licenses are typically offered on a royalty free basis, and under their terms, brand companies transfer know-how related to the manufacturing, testing, and handling of branded drugs, leaving the generic company to control local distribution [13]. The prices charged by Aspen were comparable to prices charged by Indian suppliers. (Indian and South African prices, respectively: $170 v. $149 for AZT, $64 v. $65 for 3TC, $67 v. $84 for NVP, & $45 v. $48 for d4T) The overall share of drugs provided by Aspen, assumed to represent the total produced under voluntary license, was approximately 9%.

### Consumer Country Patent Situation

Unlike in India, in Sub-Saharan Africa brand companies widely patented three out of the five first line drugs and the one combination of first line drugs eligible for protection based on a survey published in 2001 [14] (Table 4). These four drugs accounted for 45% of the volume of drugs studied (Table S1).

Generic versions of these four drugs were bought even in countries where they were patented (Table 4). Levels of generic purchasing in countries where compounds were patented were comparable to levels of purchasing in countries where the compounds were not patented (Table 4). In some cases (e.g. AZT, AZT+3TC), generic purchasing levels were actually higher in patent countries versus non-patent countries (Table 4). The data suggest that patents are not being strictly enforced in most Sub-Saharan African countries, and that the presence of patents has not uniformly deterred generic purchasing. The exceptions to this pattern are stavudine and efavirenz, which were each patented in one country, South Africa. The patents on these drugs appear to be effect in this relatively richer country, which procured no generic versions of either of them (Table 4).

## Discussion

Over the past few years, antiretroviral drugs have been bought in significant quantities for patients in developing countries for the first time in their 20-year history [15], largely with international donor funding. This study presents an empirical study of this procurement based on transactions of drugs for Sub-Saharan Africa reported to the Global Price Reporting Mechanism. While only a subset of the drugs bought, the data have relevance to ongoing debates about the role of brand and generic companies and how access to patented dugs can be achieved.

### First vs. Second Line Drugs

Significant differences between the markets for first and second line drugs were observed. First line drugs were provided by generic and brand companies, at relatively lower prices, whereas second line drugs were supplied almost exclusively by brand companies, at relatively higher prices. The data lend support to two explanations for the lack of competition in second line drugs–a lack of demand volume and patent barriers at the supplier level.

Second line drugs have been procured in lower quantities than first line drugs. Low volumes over which to spread fixed costs explain in part why even generic prices for second line drugs remain high. Nelfinavir, for example, the most procured second line drug in the dataset, was supplied from generic sources at an average price of $1021 vs. an average branded price of $980 (Table S1). It should also be borne in mind that generic companies generally compensate for low margins with high volumes [16]. This may explain in part why second line drugs, with their small volumes, were supplied almost exclusively by brand companies. As patients switch to second line drugs, at a forecasted rate of 6–10% per year of patient therapy [17], demand for these compounds should grow. While this should encourage greater competition from generic suppliers, other mechanisms for increasing purchase volumes, such as bulk or forward purchasing instruments, also deserve exploration.

In terms of patent barriers, certain second line drugs are eligible for patent protection in India, the most important supplier of the generic ARVs studied. Indian patent applications for tenofovir, abacavir, and abacavir+AZT+3TC have been reported [18]. Demand for tenofovir and abacavir should rise with their recent promotion to the first line regimen [9], yet the presence of patent applications generally can cast uncertainty on the generic market [19]. Recently signed voluntary licenses over tenofovir should be helpful in this regard [32].

Among first line drugs, the cheapest drugs–generics–do not appear to be consistently procured, as significant quantities of higher-priced brand ARVs were ordered. (Table 1) This may be due to a number of factors, including a lack of transparency about prices, availability issues, registration problems [19]–[20], or other nonprice factors. Further analyses should be undertaken to ensure that the most affordable drugs can actually be procured.

### Patents

The data confirms that generic versions of patented drugs are being procured in large quantities for Sub-Saharan Africa. They also suggest that patent barriers to generic supply have been avoided in various ways. Indian generic suppliers have taken advantage of the lack of patent protection for pharmaceutical drugs in India to produce over half of the ARVs in the dataset (Table 3). In contrast, the South African generic drugs in the dataset have been produced pursuant to voluntary license agreements with brand companies. Notably, prices of South African and Indian generic drugs appear to be comparable. Questions remain about the impact of the changes to India's patent law to the generic supply. As described above, basic versions of all individual first and many second line drugs–accounting for the majority of the supply studied–should be unaffected because of priority dates before the critical date of January 1, 1995. Demand for first line drugs should remain robust, driven by the needs of treatment naïve patients and the majority of those already being treated with these drugs. For drugs over which there are patent questions, however, voluntary or compulsory licenses could be used to foster generic competition.

The apparent lack of patent enforcement over certain first line drugs in selected Sub-Saharan African countries is notable. Some companies have formally announced that they will not enforce patents [21]–[22] or pursue patent protection in certain markets [21], [23]. Others, it would appear, have informally adopted such a policy.

Non-enforcement policies that in effect allow generic companies to produce drugs despite the presence of a patent in some ways achieve the intent of compulsory licenses without the use of formal licenses. Companies in turn may be more motivated in part to adopt such policies knowing that a compulsory license could issue at some point. International and national efforts to bolster the legitimacy of compulsory licenses, while at times criticized [24], deserve some credit for encouraging the access to patented medicines that has been achieved.

Non-enforcement and price discounting represent two “voluntary” measures that are taken by brand companies to reduce the price of drugs. Brand companies have generally limited price discounts to poorer countries such as those in Sub-Saharan Africa [25]. As such, middle-income, “producer” countries such as Brazil or India, the latter of which has the most HIV-infected patients in the world [26], are generally excluded [21]–[22]. The data suggests that non-enforcement measures have also not been extended to middle income countries: patents in South Africa over stavudine and efavirenz are correlated with a lack of generic procurement there, in contrast to patents elsewhere (Table 4). This means that, outside of Sub-Saharan Africa and the poorest countries, other approaches for encouraging generic supply will likely be used. Thailand, which recently announced it would issue a compulsory license over efavirenz [27], provides an example of one such approach.

### Brand v. Generic Companies

Finally, while brand and generic companies are often placed on opposite sides of debates about access to medicines, the data show that each has made distinct, important contributions. Generic companies have overcome concerns about quality and gained the approvals necessary [28] to become the top supplier of ARVs in the dataset. Relatively freer of licensing and patent constraints, they have devised widely used fixed dose combination drugs and encouraged later collaborations between brand companies such as the 3-in-1drug atripla made by Gilead Sciences and Bristol-Myers Squibb. They have created a viable, and in many cases cheaper, alternative to brand drugs.

Brand companies have provided a substantial percentage of the drugs procured including most second line ARVs studied. They are not enforcing exclusive patent rights in Sub-Saharan Africa and have encouraged generic production by entering into voluntary licenses with generic companies. Although the adverse impact of compulsory licensing on drug innovation has often been cited as a reason not to do it, brand companies have continued to invest in developing new drugs: as of December 2006, there were reportedly 27 HIV drugs in clinical development, with work on drugs in several new classes of treatment ongoing [29].

### Conclusion

This study has considered a single point in the supply chain for HIV drugs for Sub-Saharan African countries–pharmaceutical procurement. While other points in the supply chain, particularly those related to human resource and domestic infrastructure, pose urgent challenges [30], continued attention to drug procurement is warranted as it continues to capture a large percentage of HIV/AIDS spending [31], [32].

The data demonstrate that drugs are being procured from both generic and brand companies in significant quantities in Sub-Saharan Africa, and highlight the distinct contributions made by each supplier segment. In addition, they suggest that a combination of means, including a lack of product patents in India over older drugs, voluntary licenses by brand companies, and non-enforcement of patents have encouraged generic production of patented drugs. Each has its limitations, however–newer drugs are subject to patent protection in India and other supplier countries, voluntary licenses only account for a small fraction of the current procurement, and non-enforcement policies are available only at the discretion of brand companies and have been implemented selectively, excluding middle-income South Africa.

In addition, as others have noted, ARV prices are still significantly high as compared to per capita GDP and in light of limited local purchasing power [3]. At current cost levels and drug mix (Table 1), reaching all of the estimated 4.6 million people in Sub-Saharan Africa in need of ARVs [33] would cost $615M annually. (This conservative figure does not take into account the relatively higher cost of child formulation ARVs.) Assuming an 8% switching rate to second-line regimens and holding other values constant, this figure nearly doubles by 2010, due to the higher cost of second line drug prices. These figures indicate a long-term and increasing, not decreasing role for donors whose taxpayers also have a stake in the price of ARVs. Furthermore, provisions of international trade agreements, if enacted into domestic law, may make regulatory approval of generic drugs in certain countries harder by limiting access to needed test data [34], which could limit access. Continued attention to these and other issues should continue as they will only grow in importance with the planned scale up of treatment.

## Supporting Information

Table S1 Supporting Information for Table 1
(0.07 MB DOC)
