# Supplementary material for: HIV/AIDS Drugs for Sub-Saharan Africa: How Do Brand and Generic Supply Compare?
Source: PLoS One. 2007 Mar 14;2(3):e278. doi: 10.1371/journal.pone.0000278 (PMC1805689; doi:10.1371/journal.pone.0000278)
Supplement: Table S1 — Supporting Information for Table 1 (0.07 MB DOC) [file pone.0000278.s001.doc]

Table S1: Supporting Information for Table 1

| Drug Name | Volume (patient year) | % of Total Volume | Percentage Brand | Percentage Generic | Avg. Brand Price | Avg. Generic Price |
| --- | --- | --- | --- | --- | --- | --- |
| First Line ARVs | 522,517 | 96% | 35% | 65% | 277 | 114 |
| - Stavudine (d4T) +  Lamivudine (3TC) +  Nevirapine (NVP) | 109,971 | 20% | 0% | 100% | NA | 154 |
| - Stavudine (d4T) +  Lamivudine (3TC) | 7,537 | 1% | 0% | 100% | NA | 84 |
| - Efavirenz (EFV) | 58,343 | 11% | 88% | 12% | 386 | 332 |
| - Lamivudine (3TC) | 90,937 | 17% | 35% | 65% | 75 | 65 |
| - Nevirapine (NVP) | 93,661 | 17% | 45% | 55% | 443 | 69 |
| - Stavudine (d4T) | 77,514 | 14% | 30% | 70% | 66 | 47 |
| - Zidovudine (AZT) +  Lamivudine (3TC) +  Nevirapine (NVP) | 8,006 | 1% | 0% | 100% | NA | 156 |
| - Zidovudine (AZT) +  Lamivudine (3TC) | 61,847 | 11% | 50% | 50% | 239 | 185 |
| - Zidovudine (AZT) | 14,701 | 3% | 22% | 78% | 251 | 170 |
| Second Line ARVs | 18,984 | 4% | 93% | 7% | 591 | 601 |
| - Abacavir (ABC) | 1,566 | 0% | 100% | 0% | 893 | NA |
| - Didanosine (ddI) | 3,311 | 1% | 90% | 10% | 316 | 219 |
| - Indinavir (IDV) | 4,543 | 1% | 92% | 8% | 407 | 374 |
| - Lopinavir (LPV) +  Ritonavir (RTV) | 1,235 | 0% | 98% | 2% | 547 | 490 |
| - Nelfinavir (NFV) | 4,855 | 1% | 89% | 11% | 980 | 1,021 |
| - Ritonavir (RTV) | 288 | 0% | 100% | 0% | 87 | NA |
| - Saquinavir (SQV) | 170 | 0% | 99% | 1% | 973 | 1,086 |
| - Tenofovir (TDF) | 2,151 | 0% | 100% | 0% | 295 | NA |
| - Tenofovir (TDF) +  Emtricitabine (FTC) | 699 | 0% | 100% | 0% | 724 | NA |
| - Zidovudine (AZT) +  Lamivudine (3TC) +  Abacavir (ABC) | 167 | 0% | 100% | 0% | 1,241 | NA |
| Total | 541,501 | 100% | 37% | 63% | 304 | 116 |
